# On the Measurement of the Neutron Lifetime Using Ultracold Neutrons in a Vacuum Quadrupole Trap

**DOI:** 10.6028/jres.110.054

**Published:** 2005-08-01

**Authors:** J. David Bowman, S. I. Penttila

**Affiliations:** Los Alamos National Laboratory, Los Alamos, NM 87544, USA

**Keywords:** chaos, neutron lifetime, neutron trap, quadrupole trap, ultra cold neutrons

## Abstract

We present a conceptual design for an experiment to measure the neutron lifetime (~886 s) with an accuracy of 10^−4^. The lifetime will be measured by observing the decay rate of a sample of ultracold neutrons (UCN) confined in vacuum in a magnetic trap. The UCN collaboration at Los Alamos National Laboratory has developed a prototype UCN source that is expected to produce a bottled UCN density of more than 100/cm^3^ [1]. The availability of such an intense source makes it possible to approach the measurement of the neutron lifetime in a new way. We argue below that it is possible to measure the neutron lifetime to 10^−4^ in a vacuum magnetic trap. The measurement involves no new technology beyond the expected UCN density. If even higher densities are available, the experiment can be made better and/or less expensive. We present the design and methodology for the measurement. The slow loss of neutrons that have stable orbits, but are not energetically trapped would produce a systematic uncertainty in the measurement. We discuss a new approach, chaotic cleaning, to the elimination of quasi-neutrons from the trap by breaking the rotational symmetry of the quadrupole trap. The neutron orbits take on a chaotic character and mode mixing causes the neutrons on the quasi-bound orbits to leave the trap.

## 1. Introduction

The beta-decay lifetime of the neutrons has a direct impact on cosmological models as a production of light elements during the Big Bang. The isotopic ratios measured in extragalactic gas clouds can be compared to Big Bang Nucleosynthesis calculations in order to place limits on the ratio of baryons to photons in the early universe. In these calculations of the expected ^4^He/^1^H ratio, the dominant uncertainty is the lifetime of the neutron [2,3].

Beta decay of the neutron is both the simplest nuclear beta decay and the simplest of the charged-current weak interactions in baryons. The weak interaction parameters can be measured using neutron beta decay with fewer and simpler theoretical corrections than measurements using the beta decay of nuclei. The neutron beta decay rate is proportional to the quantity *g*_V_^2^ + 3*g*_A_^2^ where *g*_V_ and *g*_A_ are the semileptonic vector and axial-vector coupling constants. To extract the coupling constants from neutron beta decay measurements requires either an independent measurement of *g*_V_ and *g*_A_ or the ratio *g*_A_/*g*_V_ ≡ *λ*.

The other important goal of measuring the neutron lifetime with improved accuracy is to test unitarity of the Cabibbo-Kobayashi-Maskawa (CKM) matrix. For the unitarity the sum of the squares of the first row CKM matrix elements must be one

$${\left|V_{\text{ud}}\right|}^{2}+{\left|V_{\text{us}}\right|}^{2}+{\left|V_{\text{ub}}\right|}^{2}=1.$$

Experimental constraints on the values of *λ* and |*V*_ud_| are coming from nuclear 0^+^ – 0^+^ beta decays [4], CKM unitarity (assumes conserved vector current, CKM unitarity, and values of |*V*_us_| and |*V*_ub_| from [3]), measurements of the neutron beta decay correlation *A* [5]

$$\begin{array}{l} \lambda =-1.2735\pm 0.0021\\ \left|V_{\text{ud}}\right|=0.9756\pm 0.0005. \end{array}$$

In order to improve the determination of |*V*_ud_| beyond the precision of obtained from the above systems, one can study the neutron beta decay. An advantage of the study of the neutron system is the relatively stronger understanding of the necessary corrections. The nucleus dependent radiative correction δ_R_ has been calculated to the 10^−5^ level for the neutron [6], and there is no Coulumb correction. Thus, the limiting theoretical uncertainty on |*V*_ud_| determined from neutron decay is simply that due to the inner radiative correction, *Δ*^R^_V_(± 0.0004) [4]. The overall radiative corrections to the neutron decay rate have been calculated at the 10^−4^ level [6,7]. At this level, the neutron beta decay rate can be written as [7]

$$\Gamma =0.1897{\left|V_{\text{ud}}\right|}^{2}\left(1+3\lambda^{2}\right)\left(1+0.0739\pm 0.0008\right)\times 10^{-3}s^{-1}.$$

The CKM matrix element |*V*_ud_| can thus be determined from the neutron system by measuring the neutron lifetime (1/*Γ*) and *λ*. The ratio *λ* is most precisely determined from measurements of the electron-neutron spin-asymmetry coefficient *A*. Currently the limiting uncertainty on |*V*_ud_| measured with neutrons comes from *A*. As measurements of the electron-spin correlation, *A*, and the electron-neutrino correlation, *a*, are improved [8], towards the goals of proposals to measure them [9], the neutron lifetime will limit the accuracy of CKM unitarity tests. A measurement of the neutron lifetime with an accuracy of 10^−4^ will be more than sufficient for astrophysics and for CKM unitarity tests. The accuracy of the measurement will challenge the theory of inner radiative corrections.

## 2. Vacuum Quadrupole Trap

**Fig. 1:**
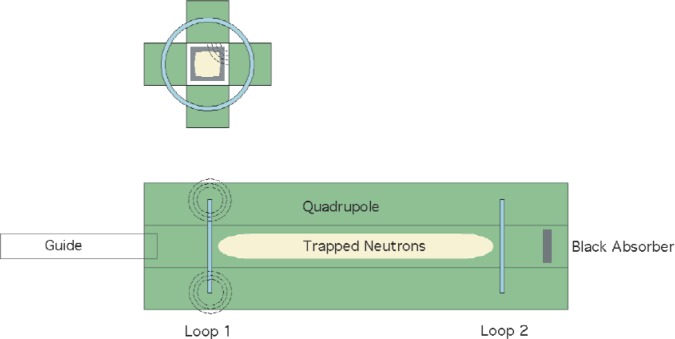
Proposed trap geometry.

**Fig. 2:**
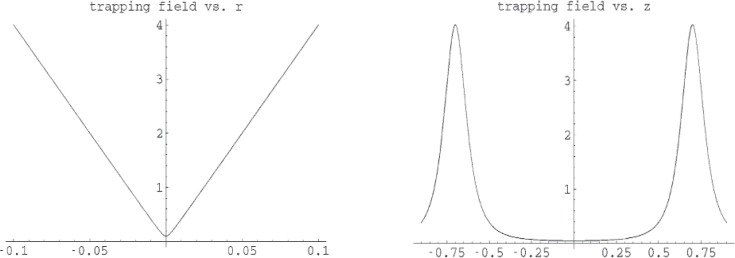
Trapping field, |*B* | in tesla, versus the axial coordinate, *z* in meters, and the radial coordinate, *r* in meters.

Figure 1 shows schematically the proposed quadrupole trap geometry and Fig. 2 shows the trapping field.

The UCNs have velocities of up to 5 m/s and the strength of the trapping field is 2.2 T. The projection of the neutron magnetic moment on the magnetic field direction is an adiabatic invariant. For fields of the order of tesla, depolarization is negligible, 10^−25^ in 10^4^ s. The above potential has minimum of |*B*| and one spin state is trapped and the other expelled. Lowering current in loop 1 fills the trap. The material guide delivers UCNs into the trap. In a few seconds the neutron population in the trap comes into equilibrium with the flux from the guide and the current is increased to close the trap. The neutrons move in the vacuum of the warm bore of the superconducting quadrupole magnet. Once the trap is closed there are no losses from the trap other than neutron β decay and the possible loss of quasi-trapped neutrons discussed below.

The above trap has been designed to have a low symmetry for two reasons. First, a low symmetry reduces the probability of quasi-trapped orbits by inducing mode mixing of the neutrons so that neutrons reach the boundary defined by their kinetic and potential energy. Second, the approximately square trap cross section helps to match scintillation detectors to the trap shape. Neutron decays are observed by detecting the up to 0.78 MeV β particles from neutron decay.

**Fig. 4:**
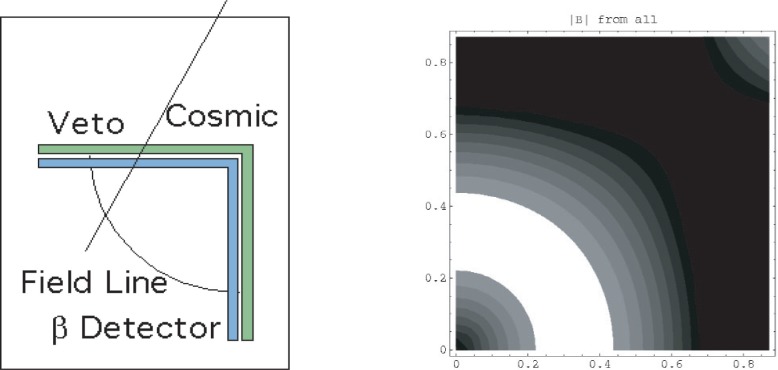
Detector scheme. Four corner-shaped scintillation (blue) detectors cover the four corners of the trap. Veto scintillators (green) to reduce cosmic ray backgrounds surround the detectors. We estimate that the veto inefficiency will be less than 10^−3^ and the cosmic/signal ratio will be 1/10. The βs spiral around field lines and are guided to the detectors. About 20 % of the βs that strike a detector will be reflected. These back-scattered βs again follow field lines and strike the other side of the same detector. The four veto detectors and the other three scintillators form an effective veto of cosmic rays and other backgrounds. On the right are shown the contours of constant yield from the trap. The density of trapped neutrons is high in the center of the trap, but the magnetic pinch effect prevents βs from these neutrons from reaching the detector. There are few neutrons trapped near the outer edges of the trap. Most detected βs come from the mid-field portion of the trap. About 30 % of the trapped βs are detected. The horizontal and vertical units on the right-hand contour plot are one graph unit = 0.1 m.

The trap geometry is chosen to facilitate the detection β particles. When a neutron decays in the trap, the emitted β spirals around field lines. Since the field lines end on the quadrupole pole faces the βs are guided to the poles. Cosmic-ray events are vetoed by the veto scintillators shown in Fig. 4.

## 3. Elimination of Quasi-Bound Neutrons

**Fig. 5:**
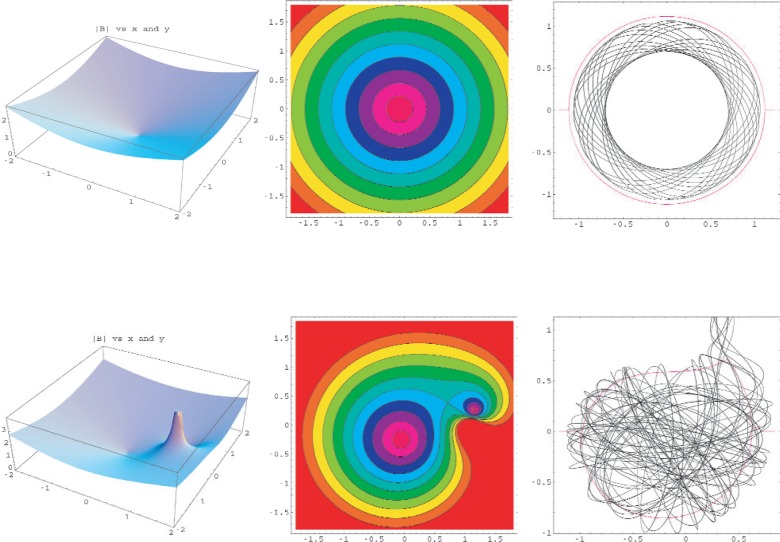
The upper row shows a symmetric quadrupole potential. The left plot shows trapping field, |*B*| in tesla versus *x* and *y*. The spatial dimensions on all plots are one graph unit = 0.1 m. A quasi-trapped orbit is shown in the right plot. The lower row is for the same quadrupole with a line current added to break the symmetry. The depth of the traps, the energy of the neutron, and the initial position and velocities are the same. The orbit crosses curve of *T* + *V* = *U*_max_ (between light blue and light green on the contour plot and the red line on the orbit plots) for the lower chaotic trap but not for the upper symmetric trap.

We have studied two approaches to the elimination of quasi-bound orbits in a two-dimensional (*x-y*) trap. Both approaches work by breaking the symmetry of the trap in a way that causes the neutron orbits to take on a chaotic character. Quasi trapping occurs when the energy of the neutron is shared between modes. The kinetic energy of the neutron never becomes zero and the neutron never reaches the surface *U* = *U*_max_. In the first approach, shown in Fig. 5, we add a line current in the *z* direction to the two-dimensional quadrupole trapping potential. The axial symmetry of the trap is broken, angular momentum is not conserved, and the orbits take on a chaotic character.

In the second approach, we insert a ^58^Ni mirror that reflects neutrons. The mirror may be inserted like a knife so that the energy added to the neutrons that collide with the mirror while it is being inserted is small. Between collisions, neutrons move in orbits having constant angular momentum. When a neutron collides with the mirror, its angular momentum changes. If the angular momentum falls below some critical value, the neutron crosses the circle of *T* + *V*=*U*_max_ and is removed from the trap.

Quasi-bound orbits may be eliminated by adiabatically reducing the strength of the trapping field, however during this procedure a large fraction of energetically trapped neutrons are lost along with the quasi-trapped neutrons. In the above example the field strength must be reduced by a factor of 8/27, and 71 % of the energetically trapped neutrons would be lost. On the other hand, the time required for chaotic cleaning of the quasi-bound orbits increases as the ratio of the neutron energy to *U*_max_ approaches unity. The best approach may be to first clean the trap chaotically for a few seconds and eliminate orbits with *U* > (1 + *ε*)*U*_max_ where *ε* ≈ 0.01. Then the quasi-trapped orbits can be cleaned by a small field reduction (*B* → 0.97 *B*_max_). Furthermore, it may require a time larger than the neutron lifetime to substantially lower the trap field strength. The smaller field strength reduction needed for chaotic cleaning is an important advantage of chaotic cleaning.

A problem that must be addressed is the activation of the guide and other objects when the trap is filled. If the act of filling the trap produces radioactivity with a lifetime comparable to the neutron lifetime, and the decays are detected, a systematic uncertainty in the measured lifetime will result. The trap and guide system will be designed to minimize activation, but the background must be measured *in situ.* The following procedure will be used to mitigate this effect. First fill the trap as if to begin a measurement cycle, but then lower loop 2 and allow the trapped neutrons to escape into the black absorber. Then restore loop 2 and measure the background.

We estimate that 8 × 10^4^ β decays are detected per fill in a 27 L trap. The neutron lifetime could be measured with a statistical uncertainty of 10^−4^ in 34 days with an UCN density of 100/cm^3^. If a higher UCN density were available, the measuring time could be shorter or the high density could be used to reduce the size of the trap, the field strength, or in other ways.

## Figures and Tables

**Fig. 3:**
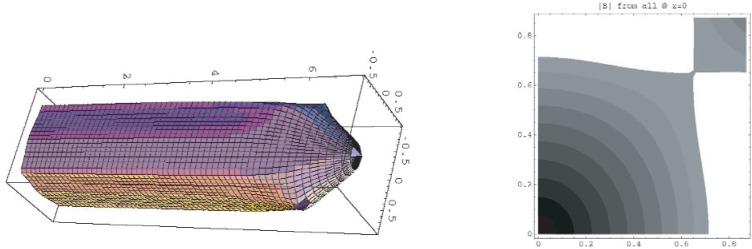
The surface of |*B*| = *B*_max_ or *U* = *U*_max_ for the quadrupole trap and the contours of |*B*| at the center of the trap. The neutrons escape along the ridge at the top of the trap—the saddle point on the contour plot—or at the cusps at the ends of the trap.

**Fig. 6:**
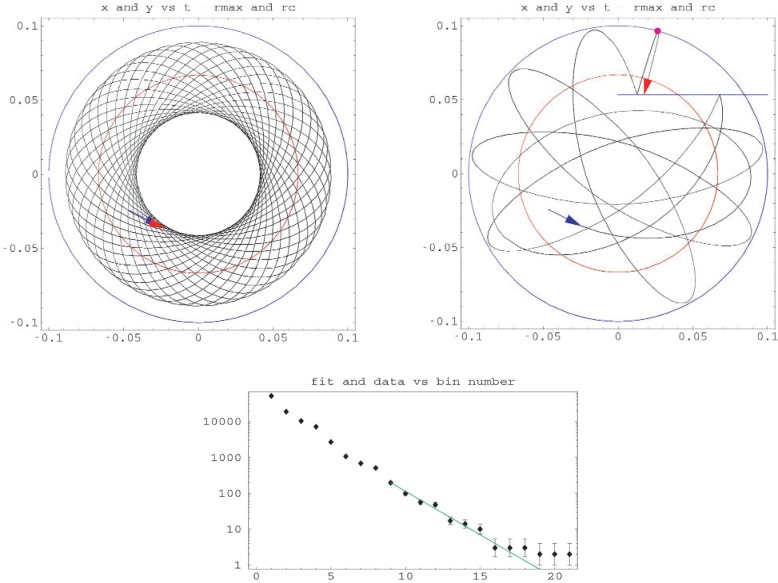
Upper two plots show trajectories in a two-dimensional quadrupole trap. The units for the horizontal and vertical axes are meters. The left figure shows the motion of a neutron with energy 1.02 times *U*_max_. The neutron never reaches the (blue) circle *T* + *V* = *U*_max_. When a (blue) mirror is inserted as shown in the upper right figure, the angular momentum is modified by collisions, and the neutron orbit crosses the circle after a few periods and is removed from the trap. The lower graph shows the distribution of removal times. The size of the time bins for the *x*-axis is 0.3 s. The approximately exponential part of the distribution has a time constant of 0.54 s. The time required for the quasi-trapped population of neutrons to be reduced to 10^−5^ of its initial value is 6.2 s.

## References

[1] Morris CL (2004). Phys Lett.

[2] Lopez RE, Turner MS (1999). Phys Rev D.

[3] Groom DE (2000). Euro Phys J C.

[4] Towner IS, Hardy JC, Herczeg P, Hoffman CM, Klapdor-Kleingrothaus HV (1999). The current status of *V*_ud_, in Physics Beyond the Standard Model. World Scientific.

[5] Abele H (1997). Phys Lett B.

[6] Wilkinson DH (1982). Analysis of neutron β-decay. Nucl Phys A.

[7] Garcia A, Garcia-Luna JL, Lopez Castro G (2001). Neutron beta decay and the current determination of |*V*_ud_|. Phys Lett B.

[b8-j110-4bow1] 8J.D. Bowman, in this Special Issue

[b9-j110-4bow1] 9W. S. Wilburn et al., in these proceedings

